# Reprogramming movements: extraction of motor intentions from cortical ensemble activity when movement goals change

**DOI:** 10.3389/fneng.2012.00016

**Published:** 2012-07-18

**Authors:** Peter J. Ifft, Mikhail A. Lebedev, Miguel A. L. Nicolelis

**Affiliations:** ^1^Department of Biomedical Engineering, Duke UniversityDurham, NC, USA; ^2^Center for Neuroengineering, Duke UniversityDurham, NC, USA; ^3^Department of Neurobiology, Duke UniversityDurham, NC, USA; ^4^Department of Psychology and Neurosciences, Duke UniversityDurham, NC, USA; ^5^Edmond and Lily Safra International Institute of Neurosciences of NatalNatal, Brazil

**Keywords:** motor cortex, sensorimotor transformation, volitional inhibition, neurophysiology, decision making, brain-machine interfaces, neuroprosthetics, monkey

## Abstract

The ability to inhibit unwanted movements and change motor plans is essential for behaviors of advanced organisms. The neural mechanisms by which the primate motor system rejects undesired actions have received much attention during the last decade, but it is not well understood how this neural function could be utilized to improve the efficiency of brain-machine interfaces (BMIs). Here we employed linear discriminant analysis (LDA) and a Wiener filter to extract motor plan transitions from the activity of ensembles of sensorimotor cortex neurons. Two rhesus monkeys, chronically implanted with multielectrode arrays in primary motor (M1) and primary sensory (S1) cortices, were overtrained to produce reaching movements with a joystick toward visual targets upon their presentation. Then, the behavioral task was modified to include a distracting target that flashed for 50, 150, or 250 ms (25% of trials each) followed by the true target that appeared at a different screen location. In the remaining 25% of trials, the initial target stayed on the screen and was the target to be approached. M1 and S1 neuronal activity represented both the true and distracting targets, even for the shortest duration of the distracting event. This dual representation persisted both when the monkey initiated movements toward the distracting target and then made corrections and when they moved directly toward the second, true target. The Wiener filter effectively decoded the location of the true target, whereas the LDA classifier extracted the location of both targets from ensembles of 50–250 neurons. Based on these results, we suggest developing real-time BMIs that inhibit unwanted movements represented by brain activity while enacting the desired motor outcome concomitantly.

## Introduction

Neurophysiological studies conducted during the last two decades have revealed a complex representation of spatial information in the brain, including the representation of multiple motor targets (Cisek and Kalaska, 2002, 2005), sequences (Mushiake et al., 1990; Isoda and Tanji, 2004), spatial attention (Lebedev and Wise, 2001; Lebedev et al., 2004; Ikkai and Curtis, 2011), and gaze (Boussaoud et al., 1993; Baker et al., 1999; Boussaoud and Bremmer, 1999; Balan and Ferrera, 2003)—all in different reference frames, depending from which brain area neural activity was sampled (Lacquaniti and Caminiti, 1998; Cohen and Andersen, 2002; McGuire and Sabes, 2009). These representations underlie rich behavioral repertoires of advanced organisms, primates in particular, that can flexibly control their attention and motor processing to meet demanding challenges of their environments (Wise et al., 1996; Wise and Murray, 2000; Lebedev and Wise, 2002). In particular, advanced organisms can inhibit and reprogram movements once the corresponding neural planning or even the movement itself have been initiated (Matsuzaka and Tanji, 1996; Band and van Boxtel, 1999; Schall et al., 2002; Mostofsky and Simmonds, 2008; Verbruggen and Logan, 2008; Stinear et al., 2009; Mirabella et al., 2011).

An adaptive neural framework can enable the planning stages of potential movements to begin in parallel with preparations for an alternative motor plan (Resulaj et al., 2009; Cisek and Kalaska, 2010). As a result, neural representations of distinct motor plans may compete prior to movement onset in behavioral tasks with several potential targets of movement (Cisek and Kalaska, 2005; Rickert et al., 2009; Mirabella et al., 2011). Studies of reaching movements have identified populations of neurons that represent multiple potential motor plans throughout the dorsal premotor (Cisek and Kalaska, 2005; Pesaran et al., 2008; Mirabella et al., 2011), supplementary motor (Chen et al., 2010), and posterior parietal cortices (Snyder et al., 1998; Scherberger and Andersen, 2007). A bounded-accumulation model (Resulaj et al., 2009) proposes that when multiple motor outcomes are presented, neural networks prepare for the most likely upcoming movement. The network accumulates noisy evidence over time until a bound threshold is reached, at which point an initial decision is reached, which is then either reversed or reaffirmed.

In the present study, we investigated the representation of multiple potential movement targets and the specification of a change in motor plan by neuronal ensembles simultaneously recorded in primary motor (M1) and sensory (S1) cortical areas. We approached neural representation of motor plan transitions from a brain-machine interface (BMI) perspective. BMIs extract motor commands from the brain and convert them into movements of external actuators, such as computer cursors and robotic devices (Andersen et al., 2004; Lebedev and Nicolelis, 2006; Fetz, 2007; Birbaumer et al., 2008; Nicolelis and Lebedev, 2009; Chase and Schwartz, 2011; Lebedev and Nicolelis, 2011; Lebedev et al., 2011). At the current stage of the BMI field, up to several hundred neurons in the brain can be recorded simultaneously by chronically implanted multielectrode arrays (Nicolelis et al., 2003; Chapin, 2004; Churchland et al., 2007; Miller and Wilson, 2008; Lebedev and Nicolelis, 2011; Lebedev et al., 2011; Stevenson and Kording, 2011). Recording from large neuronal populations is essential because the range of information extracted from neural activity and accuracy of extraction improves with the number of recorded neurons (Wessberg et al., 2000; Carmena et al., 2003; Lebedev et al., 2005; Lebedev and Nicolelis, 2006; Fitzsimmons et al., 2009; Nicolelis and Lebedev, 2009). Notwithstanding the successes of the BMI field, signals extracted from the brain are typically noisy (Lebedev and Nicolelis, 2006; Tonet et al., 2008). BMI algorithms are usually trained to reproduce one particular behavior and do not generalize well when a transition to a new set of rules and conditions is needed (Santucci et al., 2005; Fitzsimmons et al., 2009). This is why many improvements are needed: from a significant increase of the number of simultaneously recorded neurons to the development of better extraction algorithms capable of approximating natural behaviors.

Although some work has been done on the extraction of behavioral parameters during delay intervals, during which monkeys prepare movements but withhold their execution (Musallam et al., 2004; Lebedev et al., 2008; Afshar et al., 2011), the problem of motor plan transitions has not yet been fully investigated from a BMI perspective. We examined cortical representation of motor programming in a reaction-time task in which monkeys had to rapidly reprogram their center-out reaching movements. The monkeys had been previously overtrained to move a hand-held joystick toward computer screen targets. In this study, we introduced distracting targets that flashed on the screen for a short period (50–250 ms) and triggered motor preparation on 75% of the trials. This motor preparation had to be canceled when a true target appeared at a new screen location. Both the distracting and the true targets were represented by neuronal ensemble activity recorded in the M1 and S1 cortices. We used ensemble modulations to extract target locations using a linear discriminant analysis (LDA) classifier. In addition, a Wiener filter was used to make continuous extractions offline.

## Methods

### Cortical implants

All studies were conducted with approved protocols from the Duke University Institutional Animal Care and Use Committee and were in accordance with the NIH guidelines for the Care and Use of Laboratory Animals (National Research Council et al., 2011).

**Figure 1:**
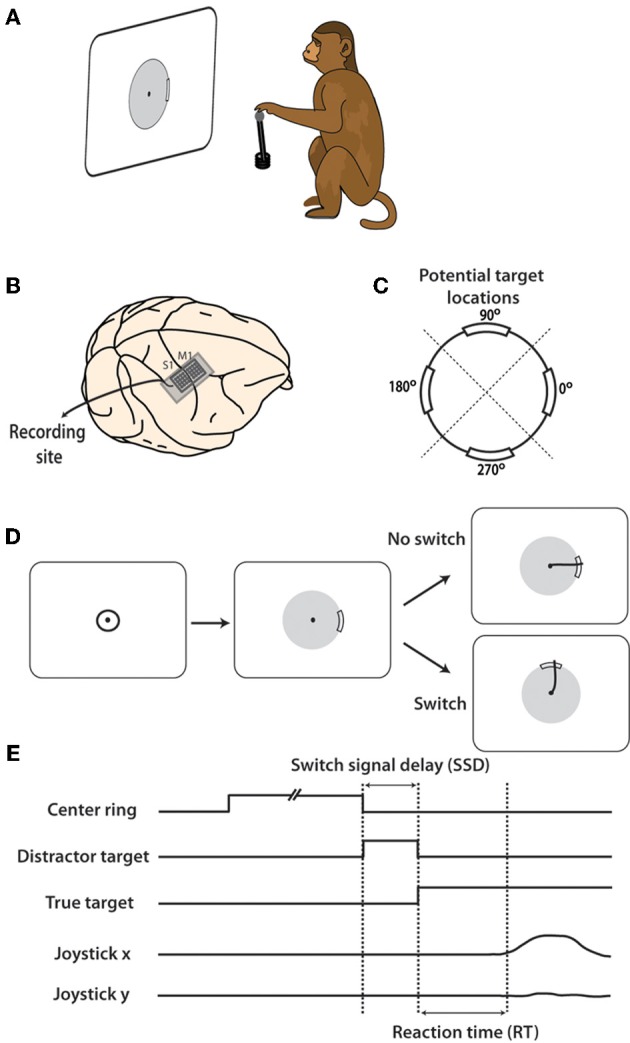
**Experiment and location of neural recordings. (A)** Rhesus monkey controlled joystick with left hand which translated to movements on computer screen. **(B)** Electrode arrays in arm representation regions of M1 and S1 cortex were implanted prior to data collection. **(C)** During task, peripheral targets appeared at one of four potential locations on the screen: 0, 90, 180, 270°. Workspace was divided into four quadrants, centered on each target location as divided by dashed diagonal lines. **(D)** Typical task sequence begins with cursor inside central target. After a random hold time, the target appears an as an arc on the gray boundary circle. On 25% of trials, this target persists and the cursor must be move through the target for reward. On the remaining trials, the target moves after a brief delay and the cursor must be move toward the new target to obtain a reward. **(E)** Shown are timelines of the presence of each target and joystick position. SSD is defined to be the time between when the first target appears and when the target is switched to the second location.

Two rhesus monkeys (one male and one female, monkeys M and N, respectively) were chronically implanted with multielectrode arrays in M1 and S1 of both right and left hemispheres using previously described surgical methods (Nicolelis et al., 2003). Within each hemisphere, two 96 channel microelectrode arrays were placed in cortical areas corresponding to cortical representations of the arm and leg (Figure 1B), but in this study, neural activity was recorded only in the arm representation area of right hemisphere M1 (in both monkeys) and S1 (only in monkey M). Each array consisted of two 4 by 4 grids of independently movable electrode triplets. Triplets were comprised of electrodes of different lengths, in 0.3 mm intervals, which allowed us to sample neuronal activity from different depths in the cortical tissue. Recorded signals were amplified, digitized, and filtered by a multichannel recording system (Plexon Inc, Dallas, TX, USA). Neuronal spikes were sorted using waveform template matching algorithm built into the real-time spike-sorting software.

### Behavioral task

Each monkey was trained to move a hand-held joystick to control the two-dimensional location of a computer cursor on a screen (Figure 1A). X(left-right) and Y(forward-backward) position of the joystick were translated to X(left-right) and Y(up-down) cursor position on the screen. The joystick was affixed to the chair at the waist level of the monkey on the side of the working hand (left hand in both monkeys). During the task, the display screen was positioned in front of the monkey, at 45 cm from the monkey's eyes. The cursor diameter was 0.5 cm.

To begin each trial, the monkey placed its left hand on the top of the joystick, causing a cursor to appear on the screen. A trial was immediately canceled if the monkey removed its hand from the joystick at any time. Next, a 5 cm diameter circle appeared at the center of the screen. The monkey moved the cursor inside this circle and held it for a random interval between 1 and 2 s. After this hold period, the center target disappeared and a single peripheral target became visible. The peripheral target appeared as a thickened 40° arc on a thin boundary circle aligned on the center of the screen (Figure 1C). Reaching the target required the cursor to pass over the thickened arc from the inside of the circle, moving outwards (Figure 1D). If the cursor crossed the circle, but missed the target, trial was terminated without reward delivery. Both monkeys had been previously overtrained to perform center-out movements toward the targets, triggered by target appearance and characterized by reaction times (RTs) of 0.49 ± 0.17 s (mean ± standard deviation) in monkey M and 0.44 ± 0.18 s in monkey N (Ifft et al., 2011).

In this study we introduced target switches to produce dual target representation in the sensorimotor cortex. This design was to mimic the momentary preparations followed by changes in motor plans. In switch trials, initial targets served as distractors. They appeared on the screen and were then replaced by a second target at different locations after a short interval, termed the switch signal delay (SSD). A similar switching task was previously reported (Georgopoulos et al., 1981, 1983) with a difference that monkeys were overtrained in those studies and M1 neurons were recorded serially. Our distracting and true targets always appeared at one of four locations on the screen, at angles 0, 90, 180, or 270° relative to the center of the screen (Figure 1C). Initial targets switched in 75% of trials. The SSD for a given trial was either 50, 150, or 250 ms, with each occurring with equal probability. When the distracting target disappeared, a second target appeared at one of the remaining three potential locations. We call this second target the true target because a juice reward was obtained only by moving the cursor through this target. In the remaining 25% of trials, the first target remained on the screen throughout the trial and a reward was obtained by passing the cursor through this target location. Once the true target appeared, the monkey had 2.5 s to complete each trial before timing out. The experiment was repeated over two daily recording sessions in both monkey M and monkey N.

**Figure 2:**
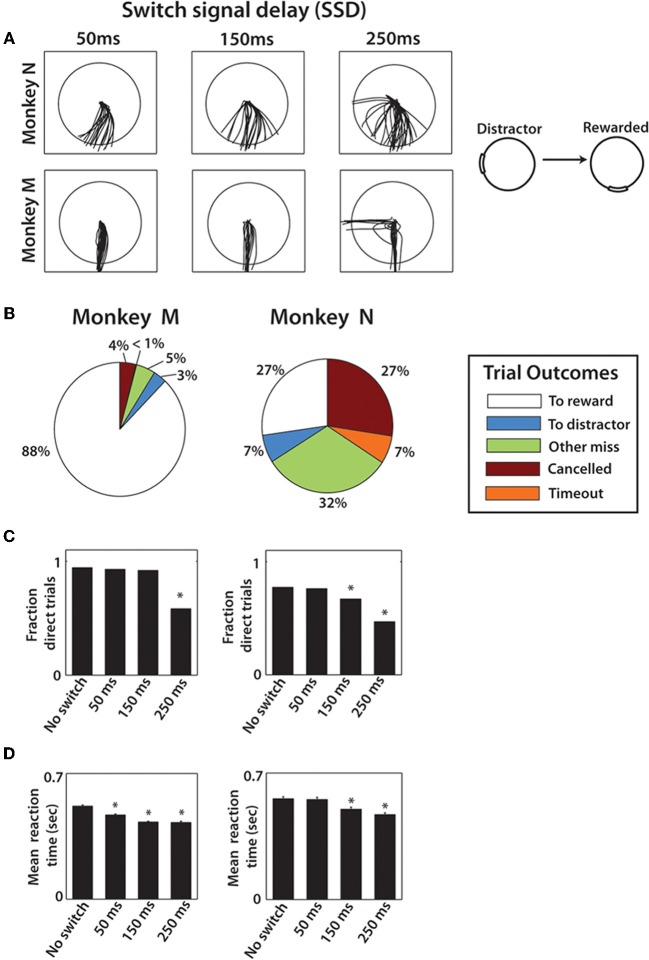
**Behavioral results from both monkeys. (A)** Typical movement traces from one combination of distractor and true target locations. The columns show different cursor trajectories on trials with different SSDs. The first row is data from monkey N and the second row from monkey M. **(B)** Pie chart shows the breakdown in trial outcomes by monkey. **(C)** The fraction of direct trials shown for each SSD group in both monkey M (left) and N (right). One-Way Kruskal–Wallis test followed by *post-hoc* unpaired *t*-tests were performed. ^*^denotes *p* < 0.001 relative to no-switch group. **(D)** Mean reaction time shown for both monkeys for each SSD group with error bars that represent standard error. Reaction time is defined as the time from true target appearance to movement onset. Same statistical procedure as **(C)**.

Single trial trajectories were categorized depending on the degree of deviation made toward the distracting target. For switch trials, a threshold distance was set at 1.5 cm along the axis between the center target and the distractor. Joystick movements which surpassed this threshold in the direction of the distractor were categorized as distracted trials. Remaining trials were categorized as direct if, in addition to not moving toward the distractor, the path to the true target deviated less than 1.5 cm in the direction orthogonal to the ideal trajectory. Strict criteria were enforced for direct trials to ensure that the only movement made was to the true target, isolating the role of movements with a singular goal from the onset. Direct trials could, however, be unrewarded if they were near misses and the cursor did not move toward the distractor. Violation of these criteria on a given trial resulted into classification as a distracted trial and later analyses evaluated these two trial groupings separately. Furthermore, trials where the initial center target was held but no movement was made to any target (beyond the 1.5 cm threshold distance) were not considered in the present analysis. Trial movement onset was computed using a previously implemented algorithm where movement initiation was detected based on the analysis of specific patterns in velocity and acceleration (Ifft et al., 2011). To perform statistical testing on performance accuracy measured as proportion of trials, such as fraction direct trials (Figure 2C) or fraction correct for different target location, trials were subdivided into groups of 15 trials. Number of outcomes of each type per 15 trials was computed for each group, and their statistical sample for all groups was entered in an appropriate statistical test (e.g., unpaired *t*-test that could be used either directly to compare two outcomes, or *post-hoc* following analysis of variance for comparing several outcomes).

RT was defined as the time from either the distractor or true target onset until movement onset. The RT measured from distractor onset was elongated by the SSD during which an initial, false, target was presented. At the same time, it could be additionally shortened for some SSDs because the distractor primed the appearance of the true target. That is, when the true target was presented, a shorter response latency could indicate that the transient distractor presence expedited pre-movement processes with respect to the true target. For the RT measured from true target onset (Figure 1E), the priming effect of the distractor is more clearly demonstrated. Each definition yields its own interpretation thus we included both in our analysis.

### Population responses

All neural analyses were performed using recordings from Monkey M M1 and S1 neurons, and Monkey N M1 neurons. Population-level analyses were conducted separately for each of these three cortical areas. Neural activity was analyzed using peri-event time histograms (PETHs) (Awiszus, 1997) aligned on distractor target onset. Recorded timestamps of action potentials were counted in bins of 50 ms width. The PETH from 0.5 s before to 1.0 s after distractor target onset (true target onset in the case of no-switch trials) for each neuron was computed separately for each combination of distractor and true target location (four no-switch combinations and 12 switch combinations), for each of the four SSD conditions. For single neuron analysis (Figures 4, 5), single trial spike rasters were constructed over the same 1.5 s epoch aligned on distractor onset. Corresponding PETHs represent the bin counts of total spikes that occurred within each of the 50 ms bins, summed across trials in the same SSD category as well as matching the same combination of distractor and true target locations. Bin counts were then divided by the fixed 50 ms bin width to represent firing rate in units of spikes/second.

To analyze population-level modulations, the average modulation profile for each neuron was normalized by subtracting the mean bin count of the neuron (over all conditions) and dividing by the standard deviation of the neuron's bin count. This normalized quantity represented modulations as a fraction of the overall modulations, or statistically, the *z*-score. The directional tuning of each neuron was computed from normalized PETH data on trials where there was no switching of the peripheral target. The mean normalized firing rate was computed within the 750 ms window following target onset for each target direction. The four directions were then ranked from most preferred to least preferred in subsequent analyses reflecting the directional preference of the each neuron (Figure 6A). Next the mean PETH over the entire population of neurons was computed for each of the sixteen distractor/true target configurations and for each of the three SSD groups (Figure 6B).

To further understand the neural representation of the distractor target and the true target, the population mean firing rate (MFR) was computed during different epochs for each of the 12 distractor-true target combinations, and four no-switch trial categories (Figure 7). Furthermore, we separated the trials by SSD to elucidate the effect of an elongated distractor presence (Figures 7A–C). For each neuron, the MFR was computed for each of the 16 positions on a 4×4 grid, with rows representing preference ranking of the true target location (ranks 1 through 4) and columns representing the preference ranking of the distractor target (ranks 1 through 4). The layout is clarified in Figure 7D. Firing rate was normalized and the directional preferences of each neuron were determined from most to least preferred direction as in Figure 6. Population MFR was obtained by averaging the MFR across all neurons in the given area. This procedure was repeated for six temporal epochs: the epoch when the distractor was present, and five consecutive 100 ms epochs following true target appearance. To evaluate the specific contribution of the distractor and true target neural representations on MFR, we fit MFR as a linear function of the preference ranking of each target preference combination (1–4) for each 4×4 grid, show in Equation 1 (Lebedev and Wise, 2001; Lebedev et al., 2004):

(1) $$\text{MFR}=A\cdot PD_{\text{true}}+B\cdot PD_{\text{distractor}}+C$$

Coefficients *A* and *B* represent the contribution of the true target and the distractor target, respectively.

We also separated trials depending on whether the monkey correctly switched to the true target (Figure 8). In this analysis, trials were separated into two groups depending on the monkey behavior: (1) trials where the true target was reached (rewarded trials) and (2) trials where the monkey was distracted (as defined earlier) and failed to reach the true target. Trials outside of these two categories were not included in the Figure 8 analysis.

To evaluate the variation in neural activity profile between the different SSD groups, single neuron normalized PETH data from each of the 12 switch conditions were subtracted from the PETH data from the corresponding no-switch condition and this value was squared. The mean of the 12 difference-squared terms was computed over the −0.5 to 1 s trial epoch for each neuron and for each SSD group, and the square root of this value was computed, yielding a root-mean-square (RMS) difference, shown by Equation 2:

(2) $${\text{RMS}}_{\text{cell}}=\sqrt{\text{mean}\left\{\sum_{i\neq j}{\left({\text{PETH}}_{jj}-{\text{PETH}}_{ij}\right)}^{2}\right\}}$$

where PETH_*ij*_ represents the normalized neural activity profile for a single neuron when the distractor is at position *i* and the true target is at position *j*. PETH_*jj*_ represents the normalized activity profile of the same neuron on a no-switch trial. Both *i* and *j* have four possible values resulting in 12 differences to be computed for each neuron. This procedure was repeated for data collected in each of the four SSD groups.

The difference profile across the population was thus computed by taking the mean difference across neurons, while maintaining temporal information (Figure 9A). Lastly, the population average for each of the SSD groups was compared to identify the relevant interval during the trial where modulations reflect the transient distractor representation (Figure 9B).

To assure that the differences arose as a result of the distractor, and not due to increased variance during elevated neural activity during movement, trials were shuffled amongst distractor location groups and the analysis was repeated, however, the SSD categorization remained intact. Once shuffled, the single neuron and population RMS differences were computed in the exact method as performed for the unshuffled data. The shuffled RMS difference profile for each SSD was generated five times and the average of these profiles was subtracted from the unshuffled population RMS difference profile, thus reflecting the true difference accounted for by the distractor presence.

### Classifier

To extract the location of both the first (distractor) target location and the second (true) target locations, LDA (Fisher, 1936) was used to decode neural activity offline and make categorical predictions of each target location (Figures 9–11) (Ifft et al., 2011). In no-switch trials, the locations of the distractor and true targets were considered the same. Neural activity aligned on distractor onset was used to train the classifier on 60% of randomly selected trials. Training data for each time point was provided by neural discharges within a 150 ms window slid across the task interval from 0.5 s before to 1 s after distractor target onset. Predictions of both target locations were made using sample data from the remaining 40% of trials. The same 150 ms sliding window was used to obtain predictions. For each session, sliding LDA predictions were computed five times, each with randomly redrawn training and sample subsets. Overall reported predictions represent the average of these five runs per session. Neural activity used to train the decoder was separated into exclusively M1 or S1 recorded neurons in the case of monkey M, and just M1 neurons in monkey N. LDA predictions were also made with shuffled data; that is, when the group information is randomly permutated prior to training the LDA classifier. For each LDA figure (Figures 10–12), we computed the fraction correct prediction of each parameter minus fraction correct of the LDA predictions from shuffled data. The chance level performance (0.25 because of four potential targets) was then added to this amount to again return to the conventional [0, 1] scale. The *y*-axis thus becomes fraction correct with unrelated modulations removed. For each analysis, confidence intervals were computed using the 1-proportion *z*-test from Equation 3:

(3) $$z=\frac{\hat{p}-p_{0}}{\sqrt{p_{0}\left(1-p_{0}\right)}}\sqrt{n}$$

where *p*_0_ is 0.25 (four potential targets) and *n* is the number of trials used for testing (40% of total trials). All confidence intervals were constructed at α = 0.05.

Trial types were then divided according whether the movement was direct to the true target or revealed a deviation toward the distractor (see Behavioral Task section above) as a way to test whether the transient representation of the distractor target is explained by motor movements. LDA was trained on both trial types combined and was then utilized to make predictions of the first and second target location separately for each trial type (Figure 11).

A separate analysis was performed using LDA to decode the presence of the switching of target location (Figure 12A). Again, a 150 ms sliding window of neural activity trained the decoder. At each time step, LDA made a prediction of whether the trial was a switch or no-switch trial. Data were again aligned on the time of distractor onset and all switch-trials were grouped together. First target in this case means distractor target in the case of switch trials, or true target (only target) in the case of no-switch trials. The 150 ms sliding window was incremented along the time axis in 25 ms steps from 0.5 s before to 1 s after first target onset. The fraction of correct predictions was computed at each time step as described for previous sliding window LDA analyses. As this analysis involves a binary classification procedure, a second metric was used to quantify extraction of event information from neural activity (Figure 12B). The Matthews correlation coefficient (MCC) is a common measure of classifiers for binary outcomes (Matthews, 1975; Baldi et al., 2000). MCC is computed as shown in Equation 4:

(4) $$\text{MCC}=\frac{\text{TP}\times \text{TN}-\text{FP}\times \text{FN}}{\sqrt{\left(\text{TP}+\text{FN}\right)\left(\text{TP}+\text{FP}\right)\left(\text{TN}+\text{FP}\right)\left(\text{TN}+\text{FN}\right)}}$$

where TP is the count for true positives predictions, TN for true negatives, FP for false positives, and FN for false negatives. Similar to the conventional correlation coefficient, the values of MCC range from −1 to 1 depending on the strength and directionality of the prediction. At each shift, the MCC was computed five times as a result of randomly redrawing the training and sample data, as was done in each LDA analysis. With two sessions per monkey, MCC at each time step of the sliding window represents the mean of 10 values.

Concurrent to prediction of trial type (switch or no-switch) at each time step, location of the first and second targets was also decoded. Although the training data included both switch and no-switch trials, LDA performance in terms of fraction correct locations only included prediction data from switch trials. This was necessary because if the no-switch target was identified as the distractor, the LDA predictions may have been artificially improved due to the prolonged presence of that target on the screen. If it was identified as the true target, the prediction may have also been improved because of the absence of interference from a distractor.

### Continuous offline predictions using wiener filter

To mimic continuous, real-time BMI predictions, we used a simple Wiener filter with six 100 ms taps of neural data to predict cursor X and Y coordinates, and true target X and Y coordinates at a 10 Hz output rate (Figure 13). For monkey M, both M1 and S1 neurons were used to improve predictions of these parameters (Figures 13A–E). To reject noisy neurons and reduce overfitting, we computed weights that reflected each neuron's contribution toward kinematic predictions. We selected the 80% of all neurons which had the highest weights.

The Wiener filter weights were fit using 60% of the session length and predictions were made using the remaining 40%. Due to variable durations of all targets in the session, we inflated the true target duration to 1000 ms to ensure targets could be represented despite a low 10 Hz rate of prediction. To reduce the effect of noisy predictions, the predicted radius of movement, *r*, was computed at each time as shown in Equation 5:

(5) $$r=\sqrt{X_{p}^{2}+Y_{p}^{2}}$$

where *X*_*p*_ and *Y*_*p*_ are the predicted X and Y position of the cursor at a given time (Figure 13B). A threshold for *r* was chosen at 5 cm (screen coordinates) such that when *r* surpassed this threshold, a reach had been predicted. Time of threshold crossing was thus recorded and predictions of target locations relative to this time were made. At each time from when *r* exceeded *r*_threshold_, predictions of cursor and true target position were made and compared to actual. To quantify performance in terms of fraction correct, the screen was divided into four (90°) quadrants surrounding each target (see Figure 1C). For example, quadrant 1 would occur from −45° to 45° relative to the center of the screen. A prediction of cursor X and Y was correct if the Wiener prediction was in the correct quadrant as the actual cursor (X, Y). True target location predictions were evaluated in the same way. Predicted true target quadrant was compared with the target locations. The fraction correct was computed at each time step beginning at threshold crossing until 800 ms after threshold crossing, during all threshold crossings in the last 40% of each session. If during this 800 ms window, *r* became less than *r*_threshold_, predictions were no longer made. If *r* was greater than *r*_threshold_, but the true target was not on the screen (with all durations fixed at 1 s, as stated before), this was counted as incorrect.

## Results

### Behavior

While the monkeys had been previously overtrained in the single target task, no prior training was performed in the task with switching targets. We chose to avoid excessive training on the two target sequence because we wanted to obtain the maximum effect of the switching target and avoid the monkeys developing alternative behavioral strategies, such as timing their responses in a way such that the initially presented target is wholly ignored. We suppose that previous overtraining in reaction-time responses toward single targets helped to enhance the representation of the distractor target because that was the target toward which monkeys were accustomed to moving in a RT manner. However, neither the contribution of prior training nor the effect of continued training with switching targets were examined in this study.

As it would be expected, the introduction of switching targets resulted in erroneous responses on a portion of trials (Figure 2), more so for monkey N. We consider reaching movements that crossed into the true target to be correct trials. Incorrect trials consisted of movements that crossed distracting targets, movements that missed both targets, canceled and time-out trials (Figure 2B). The overall proportion of correct trials was higher in monkey M than in monkey N: 88% in monkey M and only 27% in monkey N (*p* < 0.001; Wilcoxon rank sum test) (Figures 2B,C). Monkey N compensated for this inaccuracy by making many more trials per recording session (1519 and 1072 in each of two sessions, respectively) than monkey M (594 and 418 trials). This difference in accuracy is seen in the example trajectories for a particular target configuration (Figure 2A). Monkey M produced straight and carefully targeted reaching trajectories, whereas monkey N's trajectories were less accurately directed and often missed the target. Monkey M made errors only for the longest, 250 ms, SSD (Figure 2C). Another observation was that 90° switch trials were less frequently direct, compared to 180° switch trials (78.4% vs. 90.3% of trials for monkey M and 55.9% vs. 70.2% of trials for monkey N, *p* < 0.01, unpaired *t*-test). In other words, the distractor had a stronger effect on the cursor trajectory when it was closer to the true target.

**Figure 3:**
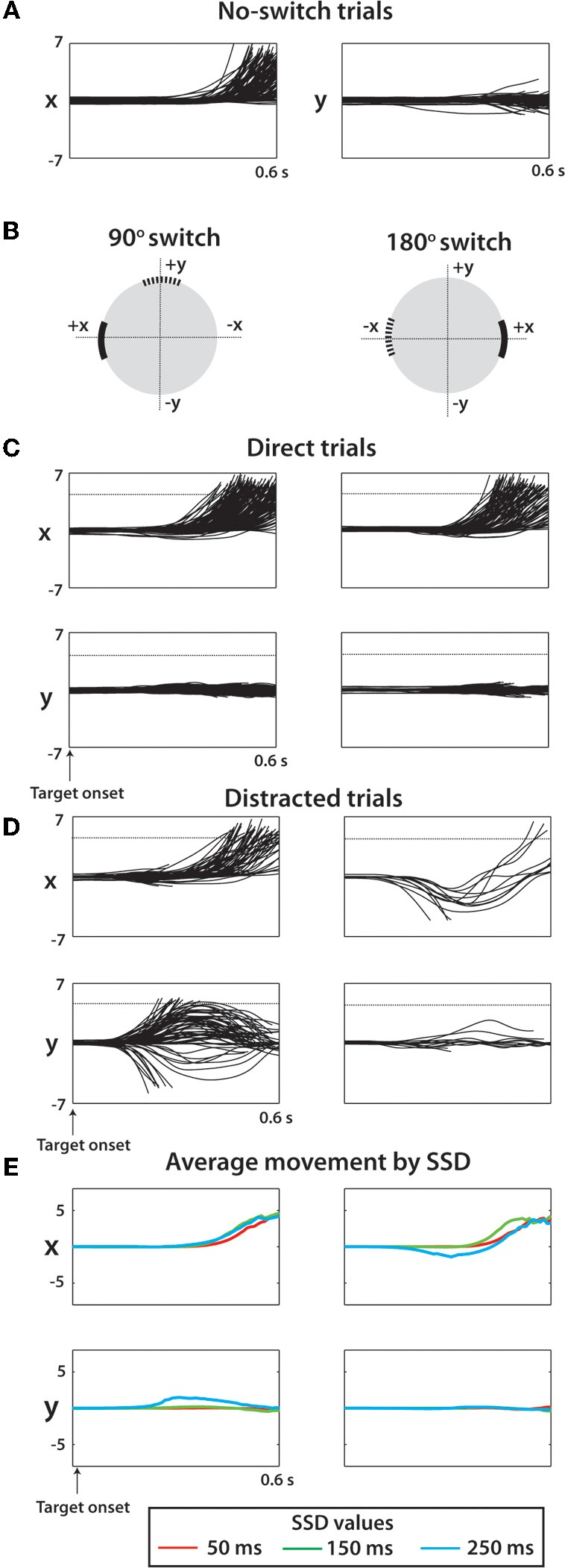
**Raw cursor trajectories for two sessions (1018 total trials) from monkey M. (A)** X and Y position of cursor versus time during no-switch trials. Offline, all targets were moved to (X,Y) position (5,0) and the associated coordinate transform was made to all kinematic data. Y indicates movement orthogonal to the ideal trajectory. **(B)** For switch trials, two categories of trials shown separately for clarity: trials with a 90° switch (left) and trials with a 180° switch (right). The coordinate systems for a given trial were rotated such that the true target was in the positive X direction and the Y direction was orthogonal to this axis. **(C)** X and Y cursor positions versus time for direct trials among 90° switch (left) trials and 180° switch trials (right). **(D)** Same as **(C)** except looking at only distracted trials. **(E)** Average X and Y trajectory of cursor separated by SSD (see Legend) and by switch angle (columns same as **C,D**).

To separate accurate and inaccurate reaches toward the true target, trials were divided into two groups consisting of direct trials and distracted trials. Classification of trials is described in detail within Methods. In the separation of all monkey M's trials, 505 trials were classified as direct, 145 classified as distracted, compared against a total of 214 no-switch trials. Joystick trajectories for these types of trials are shown in Figure 3 for monkey M. Trials are grouped by target configuration: with 90° separation between the initial and true targets (left panels), and with 180° separation (right panels). Our data show reach trajectories that deviate toward the distractor on the infrequent distracted trials. Inaccurately performed trials for long presentations of distracting targets can be also seen in the examples of Figure 2A. The trial-averaged traces reveal the largest deviation during the longest, 250 ms, SSD (Figure 3E). In both monkeys, the fraction of trials categorized as “direct” decreased with longer SSDs (Figure 2C, *P* < 0.001, Kruskal–Wallis test). More precisely, there was a decrease in fraction direct trials for 150 ms and 250 ms durations for monkey N (*p* < 0.05; *post-hoc* unpaired *t*-test) and for 250 ms duration for monkey M (*p* < 0.05) in comparison with no-switch trials. Previous studies of an overtrained switching task reported mostly distracted trials where monkeys initiated movements toward the distractor and then curved the trajectory toward the true target (Georgopoulos et al., 1981, 1983).

The effect of SSD on RT was dependent on how RT was defined (see Methods). When defined from distractor target appearance until movement onset, longer SSDs caused a lengthening in RT in both monkeys (*p* < 0.001, One-Way Kruskal–Wallis test). This is somewhat expected because with longer SSDs, the longer the monkey must wait for the true target, thus inflating the RT. However, when RT was defined relative to true target appearance (Figure 2D), longer SSD caused shorter RTs (*p* < 0.001, One-Way Kruskal–Wallis test). Thus, the appearance of the distractor on the screen primed the response to the true target, even though the directional information that it provided was incorrect. *Post-hoc* analysis revealed significant differences from no-switch trial RT among both the 150 and 250 ms SSD groups for monkey N (*p* < 0.001; Wilcoxon rank sum test) and among all three SSD groups in monkey M (*p* < 0.001). Mean RTs for monkey N (0.52 ± 0.24 s; mean ± sd) were significantly longer than the RTs for the single target task (0.44 ± 0.18 s; *p* < 0.001; Wilcoxon rank sum test), however, monkey M performance was similar in both experiments (two-target sequence RT: 0.46 ± 0.10 s; single target task 0.49 ± 0.17 s for monkey M). Overall, monkey N behavior was more erratic in the present experiment, as evidence by cursor trajectories (Figure 2A) and RT standard deviation more than twice that of monkey M. In the previously overtrained reaches to single targets both monkeys performed well (e.g., 84% and 74% correctly performed trials in monkeys M and N, respectively) (Ifft et al., 2011). Note that data from the previous study represents center-out movements to a target less than half the size of the target used in the present study.

### Neuronal responses

The initial distracting targets were represented by M1 and S1 modulations even when they appeared for a brief 50 ms interval. This representation became more pronounced with longer presentations of distracting targets.

**Figure 4:**
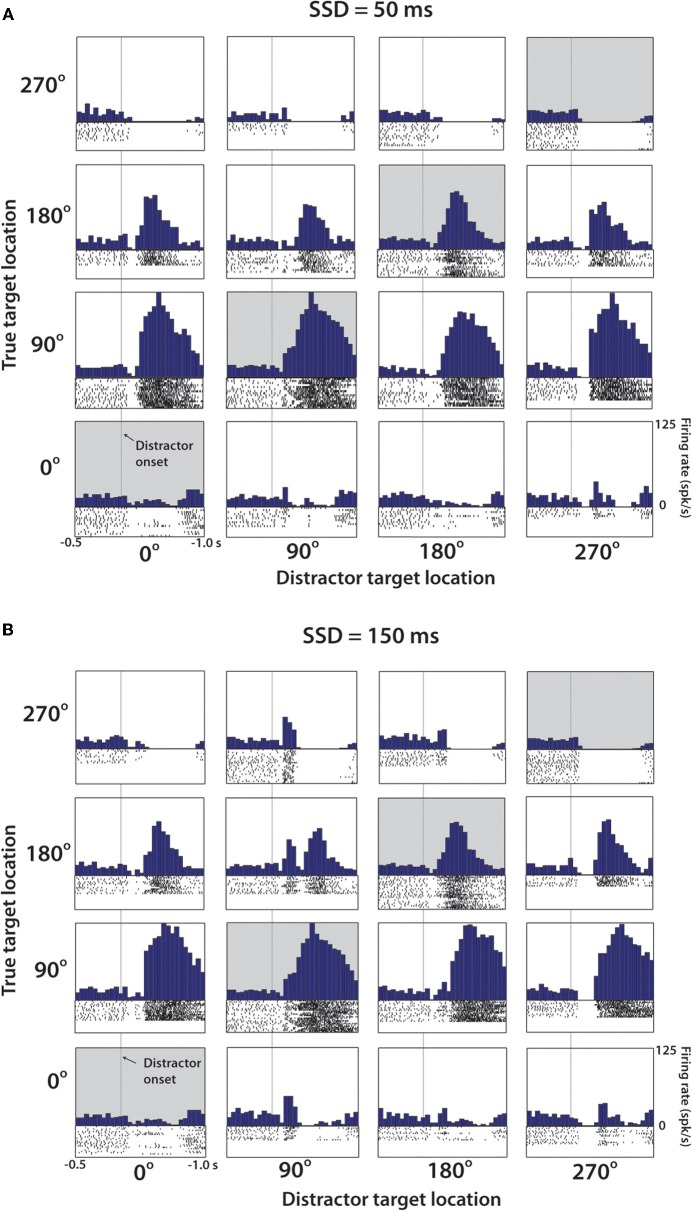
**Representative M1 neuron from Monkey M. (A)** PETH aligned on distractor target onset from trials with SSD of 50 ms. Position within the 4 × 4 grid determined by the position of the distractor and true target. Along the diagonal (shaded), these PETHs are generated from no-switch trials. Units are in terms of firing rate, where the bin count is divided by the bin width (50 ms in each case). Spike rasters below each histogram indicate time stamps of spikes from all trials of this particular combination. **(B)** Same cell and analysis as **(A)**, with only difference being that data collected from trials with SSD of 150 ms.

Figure 4 shows a representative M1 neuron recorded in monkey M that had a clear directional preference for the 90° and 180° target location and was modulated in response to both the distractor and true targets. Data are arranged in a 4 by 4 matrix representation (Lebedev and Wise, 2001; Lebedev et al., 2004) where columns of panels correspond to the distractor target location and rows correspond to the true target location. The panels on the diagonal (shaded in gray) correspond to trials where the first target did not disappear and was the true target to which the monkey had to move. Modulations reflecting the distractor location can be appreciated from the comparison of the responses within the same rows of panels, but for different columns. Modulations reflecting the true target location are seen within the same columns, but for different rows. With the exception of the diagonals, which are identical in both Figures 4A and B, Figure 4A shows data for the 50 ms duration of the distractor, and Figure 4B shows data for the 150 ms duration. The responses to the distractor target are mostly clear in Figure 4B where bursts of activity are seen in response to that target appearing at the preferred location (90°, less clear for 180°). Responses to the distractor target are not as clear in Figure 4A, but it still can be noticed that this neuron's rate was higher following the distractor target appearance at the preferred locations (90° and 180°) compared to the non-preferred locations (0° and 270°).

**Figure 5:**
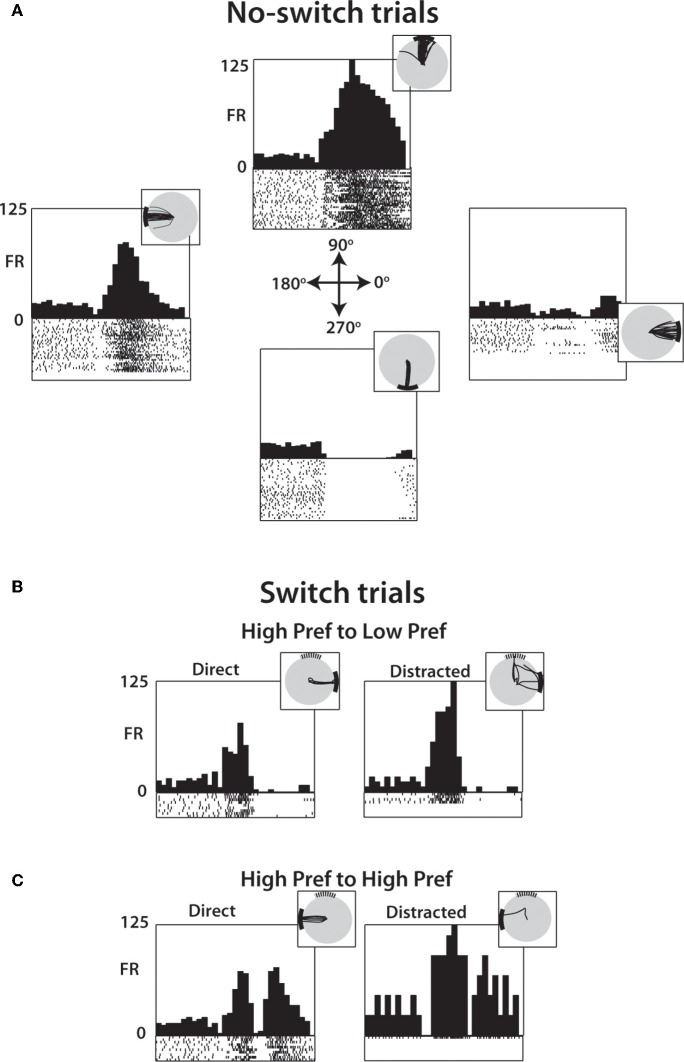
**PETH of a single M1 neuron during specific transitions after 250 ms SSD. (A)** Neural activity from no-switch trials separated by target location shows directional preferences with PETH and single-trial raster plots (below PETH) aligned on distractor target onset. Inset shows cursor trajectory from trials to the specified target. **(B)** Among switch trials, PETH and raster plots generated from trials with distractor in one of the neuron's preferred direction (90°) and the true target in a non-preferred direction (0°). Data from both direct (left) and distracted (right) shown, with inset showing cursor traces. **(C)** Same as **(B)** except data drawn from trials where both the distractor and true target are in preferred directions (90° and 180°, respectively).

Figure 5 illustrates, using the same neuron as shown in Figure 4, that the distractor target influenced neuronal patterns even when the monkey moved directly to the true target (Figures 5B,C, left panels) as it did in the no-switch trials (Figure 5A).

**Figure 6:**
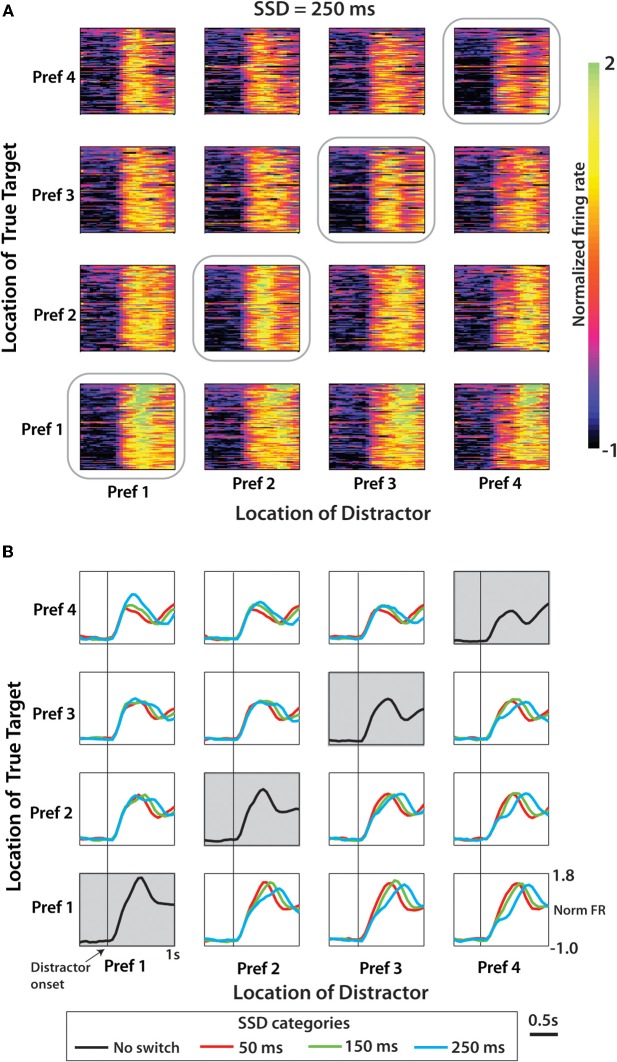
**Population activity from M1 neurons in Monkey M aligned on distractor target onset. (A)** Normalized firing rate for each cell and each pairwise combination of distractor and true targets shown for the SSD of 250 ms condition. Amplitude of firing shown by color scale (on right) interpreted as the *z*-score. Position within the 4 × 4 grid for each cell determined separately according to directional preference order. Data along the diagonal is from no-switch trials. **(B)** Population mean PETH for each of the three SSD conditions. Time-series data averaged across neurons within a specific condition (within one box on the 4 × 4 grid) organized by neuron directional preference ranking. Along the diagonal is mean population PETH for no-switch trials.

Figure 6 shows neuronal activity patterns for the entire population of M1 neurons recorded in monkey M. The format is similar to Figure 4 with the difference being that target locations were ranked for each neuron according to the firing rate exhibited for each location. Trials without distracting targets (no-switch trials) were used to rank directions into the first preferred direction (Pref 1), second preferred direction (Pref 2) and so on for each neuron. As in Figure 4, no-switch trials are shown on the diagonal (denoted by gray boxes). The off-diagonal panels show the switch trials with 250 ms SSDs. Figure 6B shows average PETHs for each SSD. Population PETHs (Figure 6A) and their averages (Figure 6B) indicate that M1 firing rates reflected both the distractor (modulations for different columns of panels with the same row) and the true (different rows within the same column) target locations. It can be also seen that each configuration of the distractor and true target locations was associated with a unique pattern of population activity. Here, as in Figure 4, the initial component of the response is modulated across panel columns (i.e., representation of the distractor target), and the late component is modulated across panel rows (representation of the true target). Average PETHs of Figure 6B indicate that the duration of the distractor was clearly reflected by the population activity—both as the average PETH shape and (e.g., bottom row of panels) and its amplitude (e.g., top row of panels). One can also notice the location of the true target was clearly reflected as average PETH amplitude for all conditions, and the location of the true target was better reflected by average PETHs for longer SSDs—both as PETH shape and amplitude. Because of these differences between PETHs for different conditions, we were able to extract information about target locations from neuronal ensemble activity using a discrete classifier.

**Figure 7:**
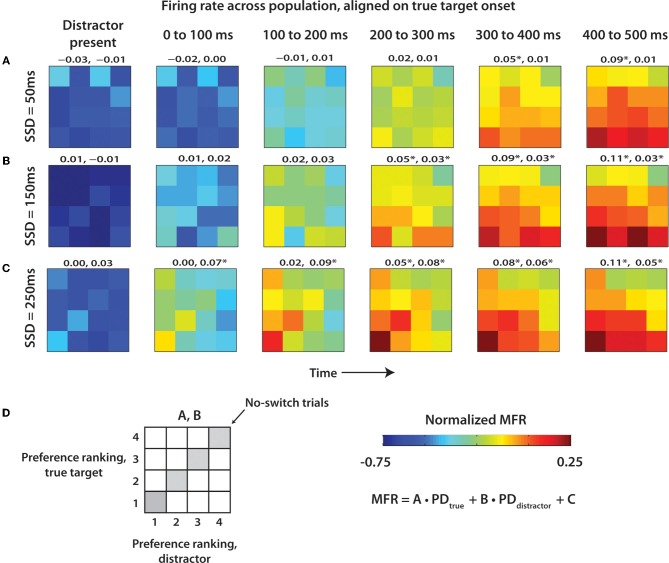
**Mean population firing rate as a function of distractor and true target locations.** 4 × 4 grids showing population MFR for each target combination. Targets for each cell were ranked in terms of preferred direction as shown in Figure 6. 4 × 4 grids were computed for epochs throughout the trial, beginning with presence of the distractor. Six epochs are reflected by six time columns proceeding from left to right. Data from each of the SSD conditions was shown separately for 50 ms **(A)**, 150 ms **(B)**, and 250 ms **(C)**. MFR within each 4 × 4 grid was fit by a linear function of true and distractor preference ranking. Coefficients for true target and distractor target, respectively, are shown above each panel. **(D)** Layout of 4 × 4 grid, color scale, and linear regression equation.

The results from Figure 6 are additionally clarified in Figure 7 that shows the evolution of neuronal rates using 4 × 4 diagrams. In the diagrams, vertical and horizontal bands correspond to neuronal tuning to the distractor and true target location, respectively. The diagrams are shown for different time with respect to true target onset (left to right) and for different SSDs (Figures 7A–C). The most prominent result seen in all SSD groups is the emergence of the true target location (bottom horizontal band of the 4 × 4 grid) as the strongest modulator of population MFR. The effect of the distractor is weaker. Early in the trial, most clearly in the 0 to 200 ms epochs, the distractor location is the primary modulator of neural activity. This is especially prominent when following a long SSD (Figure 7C). Notably, the effect of the distractor continues well into the true target presentation epoch. This is clear from the regression coefficients shown above each panel indicating the strength of the role of true target and distractor target preference ranking on MFR (see Methods). At short SSDs (Figure 7A), the distractor coefficient never reaches the level of significance (*p* > 0.05), however, the true target is strongly represented. At both 150 ms and 250 ms SSDs (Figures 7B,C), the distractor contributes a smaller, but still significant amount to the MFR. The MFR, even 400 ms after the distractor disappears, is still influenced by both targets as seen by the lower left corner triangle seen in the 400–500 ms epoch in Figures 7B,C. More generally, a transition occurs from distractor representation to true target representation.

**Figure 8:**
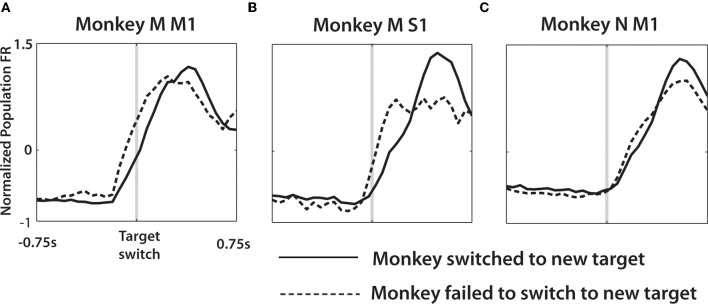
**Average population PETH differs depending on whether a switch is successfully made.** Single neuron activity was averaged across all trials where the distractor was presented. Each such trial was categorized as either a successful switch (solid line), where the true target was reached, or unsuccessful (dashed line) when the monkey failed to reach the target and the trial was categorized as distracted (see Methods). Single neuron PETHs aligned on the true target onset, for both behavioral categories were then normalized to *z*-score and averaged across the population of monkey M M1 **(A)**, monkey M S1 **(B)**, and monkey N M1 **(C)**.

Average PETHs were also used to analyze the difference in neuronal patterns between correctly and incorrectly performed switch trials. The strategy for separating trials into these two groups is described in the Methods. Average PETHs were calculated in the following steps. First, PETHs for each neuron were computed for each of 12 possible combinations of distractor and true target locations. This computation was performed separately for correctly and incorrectly performed trials. Then, PETHs for correct trials were grouped together for all combinations and all neurons, and an overall average PETH was calculated. An average PETH for incorrect trials was calculated, as well. This computation assured that the averages were not biased by the proportions of correct and incorrect trials for different conditions. We chose to average across all conditions because the differences for such averages were not specific to certain combinations of target directions. As seen in Figure 8, the average PETHs differed depending on whether the monkeys successfully switched to the true target. More specifically, trials where the monkeys switched to the true target had lower initial slopes of firing rates than trials where the monkeys failed to reach to the true target. This effect was observed in both monkeys, both in M1 and S1 neurons. In monkey M, the neural activity in both M1 and S1 clearly rose before the target switch and more steeply in trials where the monkey failed to switch to the true target. In monkey N, the difference in FR slopes was more subtle and occurred later than in monkey M, which was likely related to the more variable behavior of that monkey (Figure 2).

**Figure 9:**
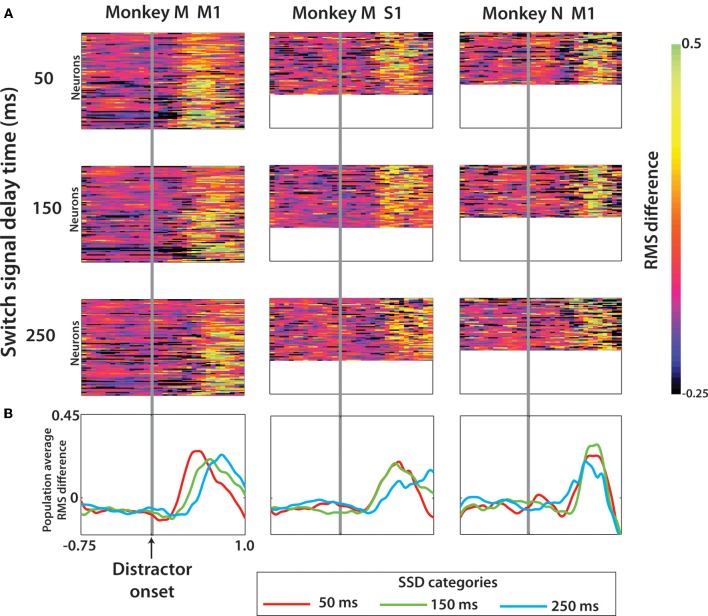
**Effect of a target switch on the firing rate in each of the three neuronal populations. (A)** RMS difference computed for each cell (see Methods) at each time step from 0.75 s before to 1.0 s after distractor target onset. Data shown separately for three SSD groups, with rows 1–3 the cell RMS difference for trials SSD of 50, 150, and 250 ms, respectively. Within each panel, the rows of the color plot indicate one single cell and the row height was fixed in all three cell groups (the three columns). The panel size is thus a reflection of the number of neurons in this population. **(B)** The population average RMS difference was computed from each panel in **(A)**.

Among rewarded trials, we analyzed neuronal representation of the target switch (Figure 9). To do this, the trial-averaged PETH for switch trials was directly compared with the PETH of no-switch trials (see Methods). As the metric for the neuronal representation of the switch, we used RMS differences between PETHs for the trials with no target switches (diagonal data in Figures 4 and 6) and the trials with target switches. Figure 9A shows RMS differences averaged across all possible locations of the distractor and true targets for each individual neuron, and Figure 9B shows the result of additional averaging across neurons in particular cortical areas. These averages depict the intervals when neuronal activity started to reflect the target switch from its initial position.

It is clear from Figures 9A and B that neurons represented target switch in M1 and S1 of both monkeys. Monkey M M1 population represented the timing of target switch for all tested SSDs, as evident from the latencies of the average curves (Figure 9B, left). S1 population of monkey M resolved the timing of the switch at 250 ms from the switches at 50 and 150 ms. The difference in switch timing was less clear in monkey N. Because of these representations of both the distracting and true targets by M1 and S1 neurons, we were able to extract target information from neuronal population activity.

### Extraction of target position with LDA classifier

**Figure 10:**
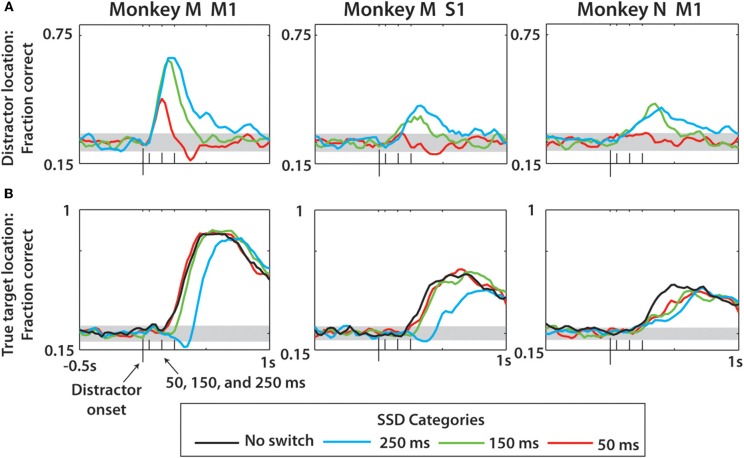
**Prediction of distractor and true target locations using neural activity and LDA classification over all trials. (A)** Prediction of distractor location for each of the three neuronal populations (columns). Within each panel, fraction correct data represents LDA fraction correct minus the same predictions with the trial data shuffled, then again adding chance level performance (0.25) (see Methods). **(B)** Prediction of true target location, which is the second target in the case of switch trials but the first in no-switch trials. In addition to three SSD groups, the no-switch prediction is shown for comparison. Gray horizontal bands indicate the 95% confidence intervals as determined by the 1-proportion *z*-test. On *x*-axis are four ticks representing the distractor onset (largest tick) and the three SSDs (50, 150, 250 ms).

An LDA classifier extracted the position of distractor (Figure 10A) and true (Figure 10B) targets from ensemble activity of M1 and S1 neurons. In the analysis depicted in Figure 10, predictions of target position were obtained from a short (150 ms) window slid along the task epoch. Behavioral trials were aligned on the distractor target onset in this analysis, and the LDA classifier was trained anew for each window position. Prediction accuracy was calculated as a fraction of correctly predicted target locations. The analysis was performed separately for 50, 150, 250 ms SSDs (red, green, and cyan traces, respectively) and no-switch trials (black trace). The LDA classifier revealed the changes in the representation of the distractor and true target locations as a function time. Note that the true target could be decoded with high accuracy despite the appearance of a distracting target. This accuracy approached 90% correct in monkey M and could be decoded nearly as fast with a distractor as without a distractor (Figure 10B). With longer SSDs, the ability to decode the true target remained similar but occurred at a longer latency. Such good decoding of the true target is not surprising given that the monkeys' overt behavior consisted of reaching movements to the true target. Future work should probe this decoding under real-time BMI control without overt behavior. The LDA model used for Figure 10 included training data from all SSD conditions, as described in the Methods. We found that limiting training data to only no-switch trials reduced the fraction correct over all SSD groups and predicted parameters in monkey M M1 by 19.9% (*p* < 0.01, paired *t*-test), monkey M S1 by 6.8% (*p* < 0.01), and monkey N M1 by 4.2% (*p* < 0.01).

Consistent with the results of Figure 7, we observed that the representations of both the distractor and true targets outlasted the duration of target presence on the screen. In particular, the representation of the distractor lasted much longer than SSD (Figure 10A). Monkey M M1 ensemble provided the best predictions of the distractor target location, as it detected SSDs as short as 50 ms. SSDs of 150 and 250 ms were clearly represented by the M1 and S1 ensembles in monkey M and the M1 ensemble in monkey N. The duration of the distractor location representation increased with the increased SSD, and for all SSDs it extended well into the true target epoch when the distractor target was turned off. The onset of the representation of the true target matched the true target onset, and the prediction accuracy was higher for the true target than for the distractor target in all cases. The peak LDA predictions for distractor location from the monkey M M1 ensemble were 20.2% more accurate than those for monkey N M1 and 34.3% more accurate when predicting the true target. To clarify whether this was the effect of different ensemble sizes, we repeated the analysis of Figures 10A,B for monkey M and N M1 populations using equally-sized subsets of each (*n* = 35 neurons; not shown in figure). We found that the prediction performance disparity in Figure 10A between the two monkeys became less pronounced (monkey M M1 now only 9.9% more accurate in predicting distractor location), but still existed, when using *n* = 35 neurons for both. The difference between the two groups in Figure 10B remained approximately the same (monkey M M1 now 33.1% more accurate than monkey N M1 in predicting true target location). From this we conclude that the size of the neuronal population was one contributing factor to LDA performance, but there were other factors as well, such as more erratic performance of monkey N and characteristics of recorded neuronal populations.

**Figure 11:**
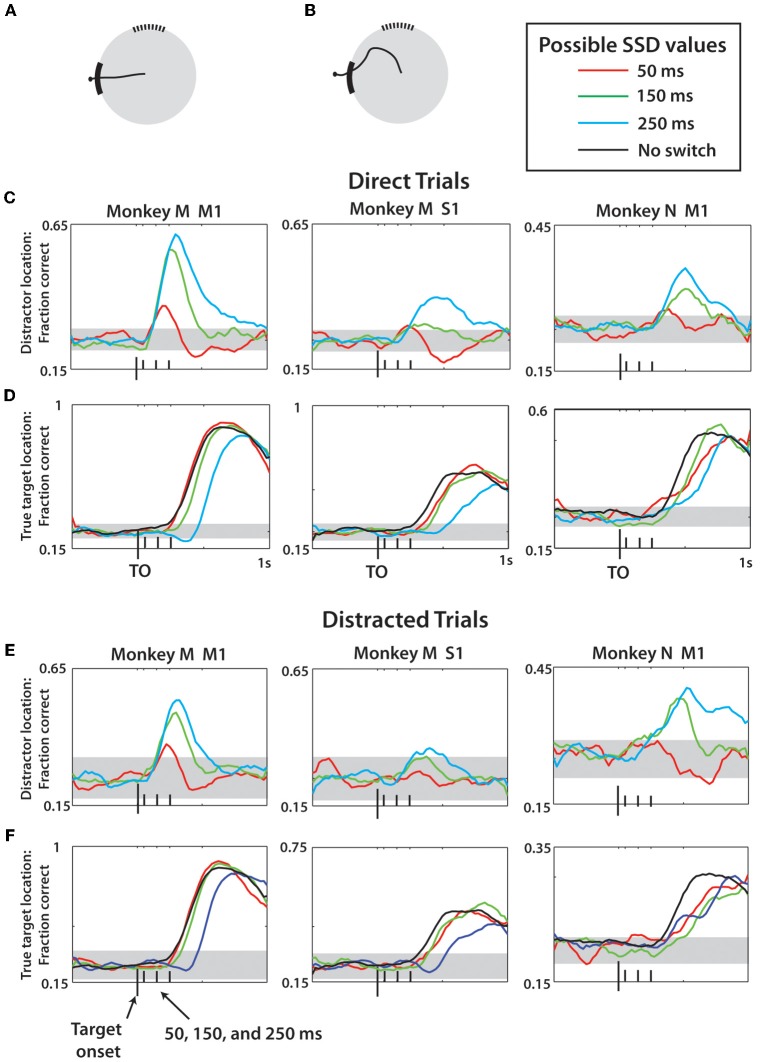
**LDA prediction of distractor and true target separated by movement type. (A)** Example of a direct trial. **(B)** Example of a distracted trial—see Methods for more details. For **(C–F)**, the prediction methods and display are the same as Figure 10. All reported data is actual fraction correct minus fraction correct from shuffled data, plus chance level fraction correct (0.25). **(C,D)** Predictions of distractor **(C)** and true **(D)** target location made using data only drawn from direct trials for monkey M M1, monkey M S1, and monkey N M1 (left to right). Different SSD groups denoted by colors, see Legend. **(E,F)** Same as **(C,D)** except data reflects only predictions made for distracted trials. Horizontal gray bands indicate 95% confidence intervals generated by 1-proportion *z*-test.

Since the predictions of the distractor target location by M1 and S1 ensembles could simply reflect the fact that in a portion of trials monkeys initiated movements to that target, we separately analyzed the trials in which the monkeys moved directly toward the true target (direct trials; Figures 11A,C,D) and those in which the presence of the distractor affected the movement trajectory (distracted trials; Figures 11B,E,F). After the direct trials were separated, the predictions remained similar to those shown in Figure 10, indicating that cortical populations represented the distractor target even when the monkey did not produce movements toward that target. Curiously, monkey M M1 and S1 ensembles predicted the distractor target location even better when that monkey made straight movements to the true target. This was likely related to the predominance of such direct trials in the training data, resulting in a better prediction model. An opposite effect was observed for monkey N, presumably because it produced less direct movements. The predictions of the true target were similar for direct and distracted trials, and similar to the predictions shown in Figure 10. Note that the predictions of the distractor location for the shortest, 50 ms, SSD were much less accurate compared to the difference plots of Figure 9. This was because the LDA training data, unlike the Figure 9 data, included all possible SSDs, as well as no-switch trials. The model had to generalize to all these conditions, which led to less accurate predictions for less represented cases. When 50 ms SSDs were analyzed separately by an LDA, the predictions improved (not shown).

**Figure 12:**
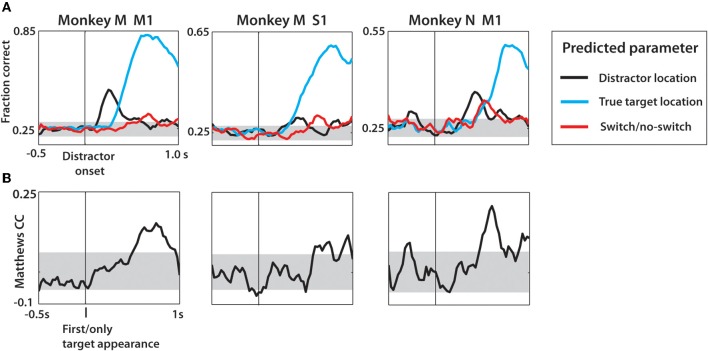
**Decoding of target location and switch/no-switch occurrence from neural activity during task interval, not separated by SSD. (A)** Linear discriminant analysis predictions of location of distractor target (black) and second target (cyan) as well as switch/no-switch (red). Data aligned on distractor onset shown with vertical black line. Values shown are fraction correct prediction at each time step of the sliding window. Gray horizontal band is the 95% confidence interval generated using the 1 proportion *z*-test. **(B)** Matthews correlation coefficient shown over the task interval. Data are aligned on the distractor appearance. Confidence interval generated from mean ± 2 standard deviations obtained from distribution of MCC from data with shuffled group information prior to LDA predictions.

In addition to the distractor and true target location, we trained an LDA classifier to predict the target switch signal as a binary variable (Figure 12). Along the task interval, predictions of the distractor and true target locations were made concurrently to serve as a temporal reference. As shown in Figure 12A, the strongest predictions made by LDA were for the true target in each case. Both monkey N and M M1 populations represented the distracting target with approximately equal facility relative to the true target representation. Overall, the predictions were less robust that those predicted when separated by SSD (Figure 10) and behavior (Figure 11). This was a result of computing the fraction correct of all three SSD groups collectively, rather than separately. This caused the less strongly predicted 50 ms SSD trials to reduce the classifier performance overall. As shown in Figure 9, the temporal profile of neural activity is strongly dependent on the SSD of a given trial. Target switch was moderately decoded in all three populations, each time with the peak occurring in the span between distractor and true target representations. The exact timing of the switch signal representation is dependent on the SSD and thus the peaks that are present in Figure 12 represent approximate event epochs. The variation on when the target switch occurs relative to the distractor onset—the time which all data is aligned to—is likely a contributing factor to the low fraction correct. If a single SSD were to be used, the switch event detection would likely occur in the SSD-dependent windows found in Figure 9B. The strongest representation of both the distractor and the switch event (note the scale difference) were obtained from monkey M M1 neurons. This is consistent with our previous data and strongly correlates with the higher number of quality recorded neural units in the Monkey M M1 population. Furthermore, the binary classification of switch vs. no-switch trial was evaluated in terms of the MCC (Figure 12B). In all three neuronal populations, the peak MCC for the switch detection occurred within 700 ms of the distractor onset, although MCC begins to rise as early as 300 ms in monkey M M1. The performance of detecting the switch event was strongest among M1 cells, with peak MCC of 0.19 (monkey M) and 0.23 (monkey N), respectively. Thus, the neural basis for motor plan switching can itself be decoded from ensembles of M1 and S1 neurons.

### Decoding of cursor and target position using wiener filter

**Figure 13:**
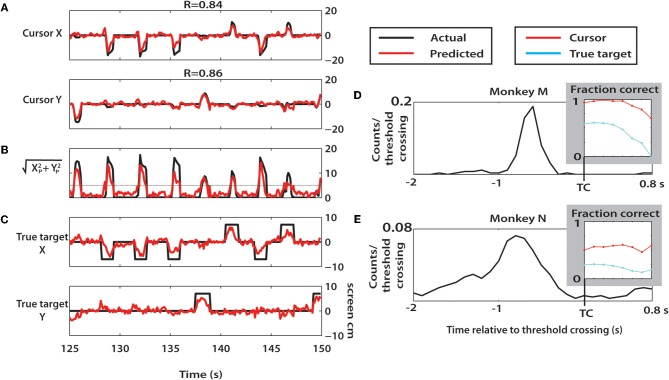
**Offline Wiener filter predictions of cursor and target locations. (A–D)** A 25 s epoch of trials during a session with monkey M. **(A)** Cursor X and Y positions (black) and corresponding Wiener predictions (red) during selected epoch. **(B)** The radial distance of the predicted cursor location was computed and plotted versus time. Reach threshold level of 5 cm is shown as horizontal gray line. **(C)** True target X and Y position during selected epoch (black) and Wiener predictions (red). **(D,E)** Probability distribution of the time of true target onset relative to threshold crossings from monkey M **(D)** and monkey N **(E)**. Inset: fraction correct predictions of cursor and true target location during the 0 to 0.8 s epoch beginning with threshold crossing.

We next utilized a continuous linear decoding algorithm, the Wiener filter, to predict cursor and true target position at a 10 Hz output rate throughout the session (Figure 13). A representative 25 s window of predicted parameters, along with actual parameter values is shown in Figures 13A–D for Monkey M. Cursor X and Y were predicted with high correlation to actual movements in monkey M (X: R = 0.84; Y: R = 0.86) and moderate correlation in monkey N (X: R = 0.49; Y: R = 0.33). Computing the predicted radial movement, *r*, resulted in clear peaks indicating predicted reach events (Figure 13B). True target location was decoded very effectively (Figure 13C). To quantify this, predictions aligned on threshold crossing of *r* were made (see Methods). We found that the threshold crossing event occurred often within 700 ms after the true target appeared in both monkeys (Figures 13D,E). Performance of cursor and true target location predictions remained consistent for approximately 500 ms after threshold crossing, before declining. (Figures 13D,E, insets). In both monkeys, the cursor position was predicted highly effectively (up to 99%, monkey M; 59% monkey N). Prediction of true target position was strong from Monkey M (up to 59%), but was much weaker for monkey N (up to 26%). Chance level prediction in this case was 20% due to four potential target locations and one condition where the target was not on the screen. The results from this continuous approach agree with the findings using a discrete LDA classifier. Furthermore, the analysis of Figure 13 provides evidence that the results of this study could be implemented into BMI systems to continuously extract intended reach locations.

## Discussion

In this study we examined M1 and S1 ensemble activity recorded in a motor task that required reprogramming of center-out reaching movements to visual targets. This was achieved by changing the target location in the midst of the RT period (Georgopoulos et al., 1981, 1983). We hypothesized that BMI decoding algorithms could dissociate representations of potential and selected motor targets from the activity of sensorimotor cortex ensembles. We found that locations of distracting targets presented shortly before the true targets of movements were indeed represented by M1 and S1 ensembles and could be extracted by an LDA classifier. The LDA results were recapitulated using a continuous Wiener filter which extracted cursor and target location. These results suggest that real-time BMI decoders could be implemented in the future to decode motor programming and decision making under the conditions of multiple potential choices.

Despite the behavioral differences between the two monkeys in this study, as is common in primate studies, both helped to elucidate behavioral responses and the neural basis for transient distractors. In our previous study (Ifft et al., 2011) we overtrained these monkeys to perform center-out movements with high accuracy when no distractor was used. In monkey N, the distractor markedly changed movement trajectories. Thus the distractor and true target locations were represented by both the overt behavior and cortical modulations. Monkey M was less distracted and the cortical effect of a change in motor plan could be studied, even when movements to the first target were wholly absent.

### Sensorimotor cortex and reprogramming movements

Neural processes of motor program selection and cancellation has received much attention during the last two decades of research. The summary of this body of work suggests that different aspects of sensorimotor transformations that involve multiple potential choices are processed by multiple cortical and subcortical areas (Crammond and Kalaska, 1994; Shen and Alexander, 1997; Lee and Assad, 2003). Here we recorded ensemble activity in M1 and S1—the areas most closely reflecting the final motor output that results from decision making. Consistent with previous work (Alexander and Crutcher, 1990) we observed M1 activity that represented potential motor targets even when no movement was initiated toward those targets. This representation persisted well beyond distractor disappearance and the termination of this encoding coincided with the onset of the robust second (true) target representation. Somewhat surprisingly, we observed moderate movement and pre-movement modulation in S1—an area whose primary function is commonly assumed to be related with sensory processing, but also known to be activated in advance of movements (Soso and Fetz, 1980; Nelson et al., 1991; Lebedev et al., 1994) and encode information about potential reach targets (Ifft et al., 2011).

Here we cannot resolve whether representation of potential targets that we observed in M1 and S1 merely reflected inputs from associative areas that were the primary players in target selection (Thaler and Goodale, 2011) or M1 and S1 constituted an integral part of a distributed network with less clearly defined hierarchy (Shen and Alexander, 1997; Hernandez et al., 2010). Visuomotor information has been shown to be encoded by cortial visual processing networks in parietal (Kertzman et al., 1997; Wise et al., 1997; Baumann et al., 2009), premotor (Crammond and Kalaska, 1994; Lebedev and Wise, 2000; Cisek and Kalaska, 2002), and prefrontal (Genovesio et al., 2005; Lebedev et al., 2005) areas. These associative areas could act as filters of sensory information that is subsequently signaled to M1 output areas. The exact mechanisms of interactions between non-primary and primary areas will have to be elucidated by future investigations.

Our previous unpublished observations indicate that certain initial stages of target selection for a movement goal have to take place for target information to start to be represented in M1 and S1. In that experiment, animals had to deal with two targets that appeared on the screen simultaneously instead of in rapid succession. One of the targets was large, and the other was small. The monkeys would be rewarded for reaching to either of the targets, but they typically selected the larger target because it was easier to hit with the cursor. In contrast to the results from our distractor experiments reported here, in the unpublished study M1 and S1 neurons represented the non-chosen target in a much more subtle way, with less than 10% of recorded cells exhibiting any significant directional tuning to its location. This observation, in the context of the results of the present study, suggests that M1 and S1 representation of movement direction is much stronger when the motor goal is chosen, even if only for several hundred milliseconds.

Serial activation of M1 during motor sequences has been well-studied (Fu et al., 1995; Tanji, 2001) and the results of our study suggest that the manifestation of change-of-decision in the motor cortex is a sequential, but somewhat overlapped representation of distinct motor plans. In other words, sensorimotor cortex represents selected motor targets, but movements to those targets can still be canceled. Such movement cannot be canceled if M1 activity is already elevated and has reached a certain motor initiation threshold (Figure 8) (Hanes and Schall, 1996; Wong-Lin et al., 2010).

A prominent model to describe the change-of-decision is a bounded form of the accumulator model (Vickers and Smith, 1985), drift-diffusion model, or race model with criterion boundaries for both initial decision and change of decision events (Resulaj et al., 2009). Applying this model to neurophysiology of sensorimotor neurons, one hypothesis would be that the firing rate of a single neuron or entire neuronal populations would encode the degree of commitment to the specific motor plan. Lower levels of activity would elongate the decision window while additional evidence is accumulated, even if a different movement had been initiated. To address this hypothesis our study compared population activity between a subset of trials where the true target was successfully reached versus trials where an error in behavioral outcome was caused by the transient distractor presence. Whether this lower population activity is causal to the behavioral differences is beyond the scope of this study. However, our results reinforce this model by showing lower initial population activity and more gradual FR slope between distractor target presentation and movement initiation on trials where the switch was successfully made (Figure 8). Such differences in population activity may provide intuitive understanding for the ability to detect the switch/no-switch event using neural activity from single trials (Figure 12B) with high fidelity.

### Decoding motor reprogramming

Here we used a rather simple LDA decoder that extracted target and target switch information from both cortical activity and the timing of the distractor target onset. This decoder was useful to describe the representation of targets by neuronal activity as a function of time. A practical decoder will have to extract target onset, as well. Our BMI approach added an interesting twist to our experiments because information extracted from different parts of sensorimotor hierarchy could be used to retrain brain circuitry. For instance, learning a BMI task that involves extraction of target information may result in an enhanced representation of such information in M1. Additionally, non-primary areas should be considered as sources of information about multiple potentials targets (Snyder et al., 1998; Cisek and Kalaska, 2002, 2005) for a practical real-time decoder. With the current approach, we were able to extract the location of distractor targets from the primary sensorimotor cortical activity even if those targets were presented for a brief period of time (as short as 50 ms) and if no movement was initiated to that target. It is important to emphasize that under this same condition, the true target to which the monkeys moved was also decoded very accurately.

As the BMI field advances, practical, versatile neuroprosthetics based on BMI technology become a real possibility (Lebedev and Nicolelis, 2006; Nicolelis and Lebedev, 2009; Gilja et al., 2011; Jackson and Fetz, 2011; Lebedev et al., 2011). The need for practical clinical applications that provide higher degree of freedom control (Velliste et al., 2008) and expanded decoding strategies (Zacksenhouse and Nemets, 2008) will drive BMI research to expand into more complex motor programs. Naturally enacted movements require the flexibility to rapidly modify upcoming motor plans. Such a behavior capability was reflected in the neuronal data we collected in the present study. The ability to decode such changes has critical implications for not only accuracy but also safety in the execution of everyday movements by a prosthetic device controlled by brain activity.

Our present experimental approach, based on a discrete rather than continuous decoder, adds to previous literature where similar ideas were evaluated under the framework of a potential cognitive neuroprosthetic (Musallam et al., 2004; Pesaran et al., 2006). A cognitive neuroprosthetic extracts from brain activity information that is different from motor execution signals and utilizes it to improve the performance. For example, a high-performance BMI proposed by Santhanam et al. (2006) extracted target location from delay-period activity recorded in dorsal premotor cortex and thereby obtained information transfer rate of up to 6.5 bits per second. Additional improvements may come from hybrid BMI designs that utilize both single-unit recordings and local field potentials (LFPs). Thus, Hwang and Andersen (Hwang and Andersen, 2009) decoded movement onset from LFPs while decoding movement direction from single-unit activity.

Hasegawa et al. (Hasegawa et al., 2006, 2009) implemented decoding algorithms that served a similar purpose that we describe here. They decoded go/no go decisions from the activity of 2–5 neurons recorded in monkey superior colliculus and were able to extract multidimensional decisions (e.g., go/no go for two potential movement directions). The information was accessed approximately 150 ms after cue onset, which is consistent with our present results and the results of Santhanam et al. (2006). Given a high interest to neurophysiological mechanisms of response inhibition (Hanes and Schall, 1995; Pare and Hanes, 2003; Chen et al., 2010; Scangos and Stuphorn, 2010; Mirabella et al., 2011), it is reasonable to expect that BMIs that extract response inhibition and response reprogramming information will continue to develop.

### Versatile BMIs of the future

The original conception of BMI systems strive to mimic normal functions of the brain as closely as possible (Nicolelis, 2001). The approach that we propose here can be generally characterized as a BMI with impulsivity control. Impulsivity is a person's inability to inhibit unwanted actions (Basar et al., 2010; Kim and Lee, 2011). Prefrontal mechanisms are normally responsible for such inhibition in primates (Miller, 2000; Krawczyk, 2002; Kim and Lee, 2011). It is conceivable that practical BMIs of the future will need an inhibition control module to operate properly. Moreover, such a module may become one of the essential elements of the design. It may not only examine potential actions and select those that fit the context and are wanted by the user, but also set the limits to volitional control. In the past, we have already proposed that such an optimal design may be based on a shared-control BMI, i.e., one that gives the user control over higher-order goals and delegates lower-order controls to the robotic controller (Kim et al., 2006). A prominent role of prefrontal cortex is executive function, such as the one required for inhibition of potential actions. Future work could seek to exploit the multiple levels of control within the brain to not only recreate naturalistic movements, but at the same time streamline the transitions and selections from the many possible behavioral outcomes. Certainly this goal is challenging, but we remain optimistic in light of recent developments in the fast growing field of neuroprosthetics.

### Conflict of interest statement

The authors declare that the research was conducted in the absence of any commercial or financial relationships that could be construed as a potential conflict of interest.
